# Comparison of Two Storage Methods for the Analysis of Cholinesterase Activities in Food Animals

**DOI:** 10.4061/2010/904249

**Published:** 2011-01-17

**Authors:** Kasim Abass Askar, A. Caleb Kudi, A. John Moody

**Affiliations:** School of Biomedical and Biological Sciences, Faculty of Science and Technology, University of Plymouth, Plymouth, PL4 8AA, UK

## Abstract

Cholinesterases (ChE) are specialized carboxylic ester hydrolases that catalyse the hydrolysis of choline esters. They are classified into either acetylcholinesterase (AChE) or butyrylcholinesterase (BChE). Determination of ChE in the tissues is the appropriate tool for the diagnosis of organophosphorus and carbamate exposures. In general, a significant inhibition was seen in both AChE and BChE activities after 6 months of freezing at −80°C and after 3 months of freezing at −20°C. Linear regression of mean AChE and BChE was observed in all individual samples during the months of the two freezing methods. Bland and Altman plot of the ratios of the two freezing methods have showen the mean difference between the two freezing methods to be 8.8, and SD was 144.7 and −127.6 for upper and lower limits, respectively, for liver, while in muscle the mean difference was 1.5 and SD was 32.5 and −28.9 for upper and lower limits, respectively.

## 1. Introduction

Cholinesterases (ChE) are specialized carboxylic ester hydrolases that catalyse the hydrolysis of choline esters. Two types of ChE activity have been identified in mammalian tissues; these are distinguished according to their substrate specificity and sensitivity to the selective inhibitors. The first is acetylcholinesterase (AChE, EC.3.1.1.7), which is systematically called acetylcholine acetylhydrolase. Other names include true cholinesterase, specific cholinesterase, red blood cell cholinesterase, erythrocyte cholinesterase, and cholinesterase I. The second is butyrylcholinesterase (BChE, EC.3.1.1.8), referred to systemically as acylcholine acylhydrolase. Other names include pseudocholinesterase, nonspecific cholinesterase, plasma cholinesterase, serum cholinesterase, propionylcholinesterase, benzoylcholinesterase, and cholinesterase II [1–4].

The preferred substrate for AChE is acetylcholine (ACh), BChE prefers butyrylcholine (BCh), and propionylcholine (PCh) [1, 2, 5]. AChE and BChE serve a pivotal role in regulating nerve impulse transmission by rapid hydrolysis of the neurotransmitter ACh [6, 7]. AChE appears to be the predominate enzyme performing this function, since AChE catalysis the hydrolysis of ACh much more rapidly than BChE [6, 8]. At present, the most widely used method for the determination of ChE activity is the colorimetric method of Ellman et al. [9]. This is a simple, accurate, fast, and direct method of measuring ChE activity in blood and tissues. It is based on the reaction between thiocholine, which is one of the products of the enzymatic hydrolysis of the synthetic substrates acetylthiocholine iodide (ATCI) or butyrylthiocholine iodide (BTCI), and the sulfhydryl group of a chromogen such as 5,5′-dithiobis-(2-nitrobenzoic acid) (DTNB or Ellman's reagent). The formation of the yellow product of this reaction, 5-thio-2-nitrobenzoic acid (TNB), is measured by monitoring absorbance at 410 nm. Each mole of the anion produced represents the hydrolysis of one mole of substrate [5, 10–12]. The advantage of DTNB is that it is water soluble; it may be used at neutral pH with few side reactions; its reaction with thiocholine is fast and sensitive due to the high molar absorption coefficient of TNB [5, 9, 13]. The objectives of this study were (a) to investigate correlations between the storage −80°C and −20°C, and (b) to establish a foundation for the applicability of ChE activities in food animal species as biochemical biomarkers for the evaluation of exposure to organophosphorus and carbamate pesticides.

## 2. Materials and Methods

### 2.1. Chemicals

Cholinesterase (ChE) substrates (acetylthiocholine iodide, ATCI, 98% purity; S-butyrylthiocholine iodide, BTCI, 98% purity), and 5,5′-dithiobis-(2-nitrobenzoic acid) (DTNB) were supplied by the Sigma Chemical Company (Poole, UK). All other reagents and solvents used in this work were of analytical grade and were supplied by Fisher (Loughborough, UK).

### 2.2. Animals

Meat from healthy food animals (sheep, cattle, and pigs) from local markets in Plymouth and abattoirs in Cornwall (Callington and Launceston), UK, was used in this study. The samples (liver and muscle) were transported on ice to the laboratory for immediate processing. During sample collection from the animal it was ensured that there was no possibility of the introduction of Anti-ChE compounds from the skin of the animals. As noted by Fairbrother et al. [14], this can be a source of contamination by Anti-ChE.

### 2.3. Sample Preparation

One gram of each tissue was removed using a scalpel, cut into small pieces (3-4 mm^3^), and rinsed until the blood was fully removed. The tissue was then placed on ice in 12 mL tubes (7.5 mm internal diameter) and homogenized using a mechanically driven homogenizer (Model X520-D, T6 probe, Bennett and Company, Weston-super-Mare, UK) with sodium phosphate buffer (0.1 M, pH 8) at a ratio of 1 part of tissue to 9 parts of buffer, and a speed of 10000 rpm. Homogenisation required between 2 and 5 min depending on the tissue; after every 30 s or so of homogenisation the mixture was rested for 10 s to allow cooling. The homogenate was then centrifuged and then decanted into an Eppendorf tubes at 9000 g for 5 min at 4°C [12, 15, 16]. However, the homogenates were thoroughly mixed and distributed into 16 equal portions in all animals (sheep, cattle, and pigs) and tissues (liver and muscle), representing storage temperatures (−80°C and −20°C) for immediate processing one-month intervals, over a period of eight months. It was important during homogenization to ensure that (i) samples were fully homogeneous and that aliquots taken reflected the homogenate as a whole, and (ii) that ChE activities were altered in the process (e.g. through heat-induced denaturation) [14].

### 2.4. Enzyme Activity Measurement

Cholinesterase activity was determined by the Ellman method [9], adapted for use with microtitre plates as described by Haigh et al. [17], and using either ATCI or BTCI as substrate (1 mM final concentration of each) for measuring AChE and BChE activities, respectively. Substrate solutions were prepared and used on the same day and kept on ice during use. Briefly, 0.02 mL of sample and 0.24 mL of assay mixture (9.75 mL of 0.1 M sodium phosphate buffer, pH 8.0, containing 1 mM EDTA, and 0.25 mL of 0.2 mM DTNB) were mixed, allowed to stand for 5 min, and then 0.04 mL of substrate solution were added. The absorbance increase was monitored for 5 min at 410 nm, at 25°C in a plate reader (OptiMax, Molecular Devices, Sunnyvale, CA) [17]. In each case the rate of absorbance increase was corrected by subtracting the rate observed for a reagent blank (i.e., without sample). ChE activities were calculated using an extinction coefficient of 13.6 mM^−1^ cm^−1^ for TNB [18]. All measurements were carried out in triplicate.

### 2.5. Data Analysis

Conventional statistical methods were used to calculate the means, coefficient of variance, and standard errors (SE). Pearson's correlation coefficient were applied to test for any significant differences (*P* < .05). All statistics were carried out using MiniTab statistical software version 15 (MiniTab Ltd., PA, USA). Bland-Altman method was also used to compare between two storage methods according to Dewitte et al. [19].

## 3. Results

### 3.1. Liver Freezing

**Figure 1:**
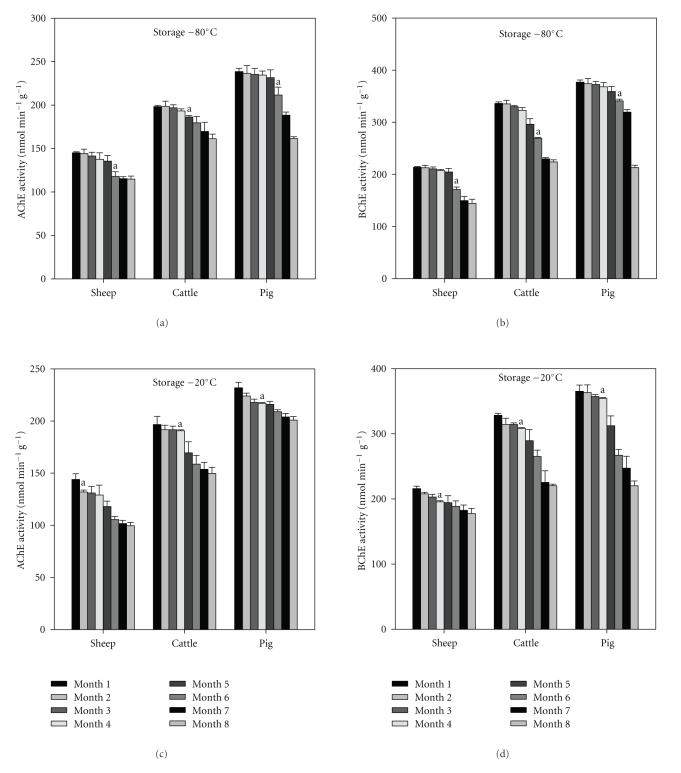
AChE and BChE activities in liver for sheep, cattle, and pigs using freezing at −80°C ((a) and (c)) and −20°C ((b) and (d)). Data are expressed as the mean ± SE (*n* = 10 of each animals). The letter in the column is significantly different (ANOVA, *P* < .05).

Acetylcholinesterase (AChE) and butyrylcholinesterase (BChE) activities were determined in liver for sheep, cattle, and pigs of each of the 8 freezing times rates at −80°C and −20°C as described in Section 2 (Figures 1(a)–1(d)). There were significantly higher AChE and BChE activities in pigs compared to when cattle and sheep used, using both freezing effects (Figures 1(a)–1(d)). In all cases (sheep, cattle, and pigs using both freezing methods), BChE activity was higher in liver than AChE activity (Figures 1(a)–1(d)). Freezing for cases (sheep, cattle, and pigs using both freezing methods) at −80°C showed a significant decrease of AChE and BChE activities after 6 months (Figures 1(a) and 1(b)). In general, freezing at −20°C showed a significant decrease in AChE and BChE activities after 3 months with an exception in the case of sheep in which the decrease in AChE was significant after 1 month (Figure 1(c)). 

**Figure 2:**
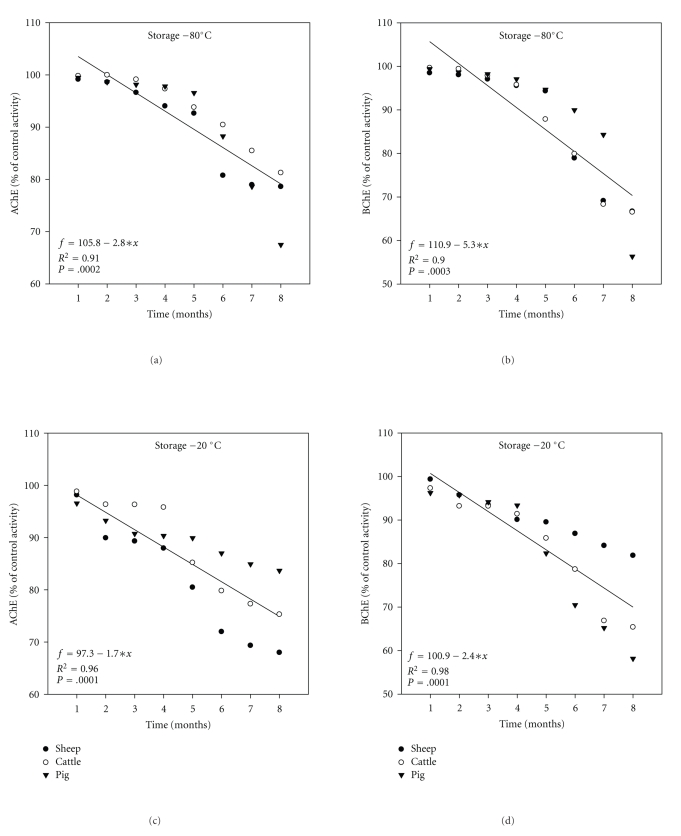
Regression analysis of ChE (% of control activities) over time in liver using freezing at −80°C and −20°C for sheep, cattle, and pigs. A linear regression equation is usually written as follows *f* = *a* + *bx*, where *f* is the predicated mean ChE activities, *a* is the intercept of the regression line with *f*-axis, *b* is the slop or regression coefficient and *x* was any month of storage. These equations indeed could be used for predication of ChE activities in different sites for any month of storage.

**Figure 3:**
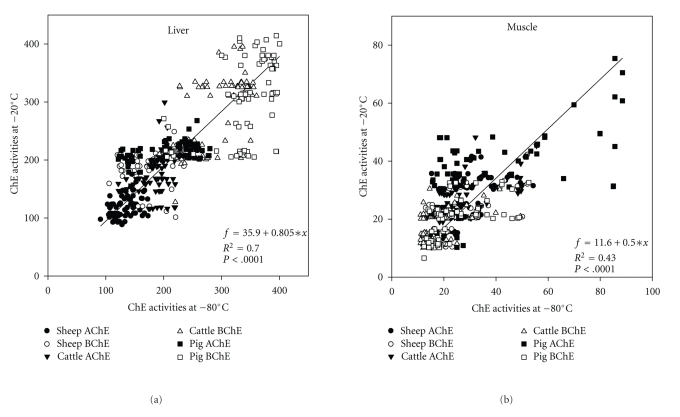
Regression analysis of individual activity of AChE and BChE using storage at −80°C and −20°C in liver and muscle of sheep, cattle, and pigs.

**Figure 4:**
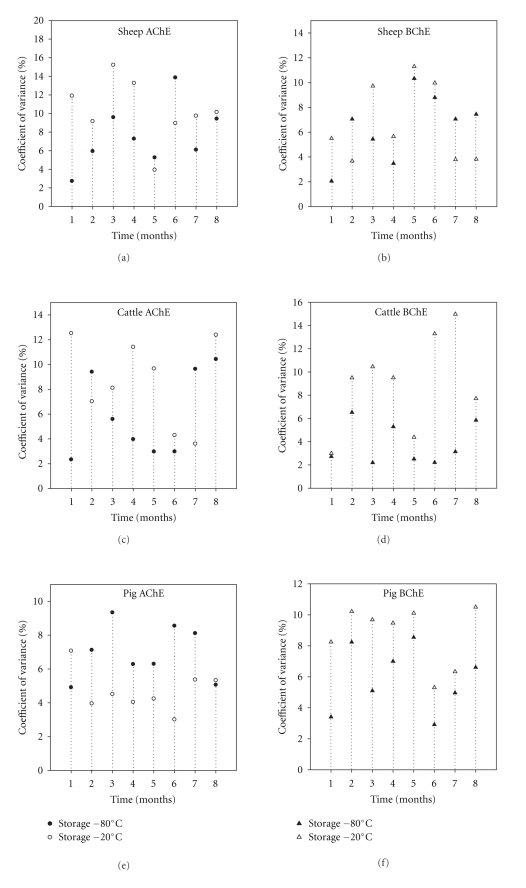
Percentage coefficient of variance between freezing at −80°C and −20°C for liver sheep, cattle, and pigs in 8 months.

**Figure 5:**
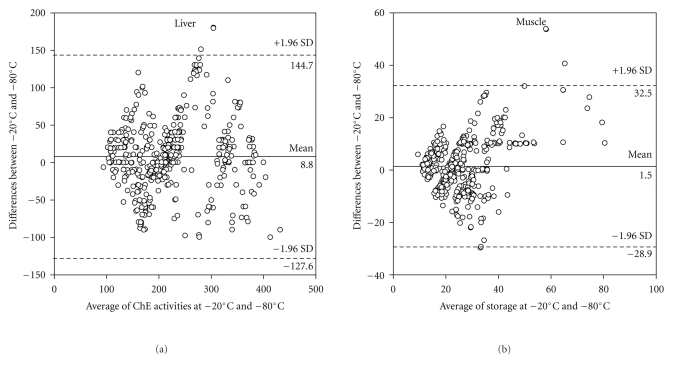
Bland and Altman plot of the ratio of the storage at −80°C and −20°C (plotted on the *y*-axis) versus the *average *of the storages (*x*-axis) for food animal ChE activities. Horizontal lines are drawn at the mean difference, and at the mean difference ±1.96 SD of the differences (dashed line). If the differences within mean ±1.96 SD are not clinically important, the two storages may be used interchangeably for tissue samples.

The linear regression of mean ChE activities of liver after 8 months of freezing is shown in Figures 2(a)–2(d). The *R* ^2^ values tended to be very high in case of BChE activity at −20°C (*R* ^2^ = 0.98, *P* = .0001; Figure 2(d)). Taking the overall data set there was a significant correlation between both AChE and BChE activities measured at −80°C and −2°C of freezing (Pearson′s correlation coefficient = 0.70, *P* < .0001; Figure 3(a)). However, the percentage of coefficient variance (%CV) values for each month was generally higher (35 out of 48 sets of data) using the −20°C compared to the −80°C freezing (Figures 4(a)–4(f)). Bland and Altman plot of the ratio of the two freezing methods at −80°C and −20°C showed the mean differences between two freezing methods to be 8.8, and SD was 144.7 and −127.6 for upper and lower limits, respectively (Figure 5(a)).

### 3.2. Muscle Freezing

**Figure 6:**
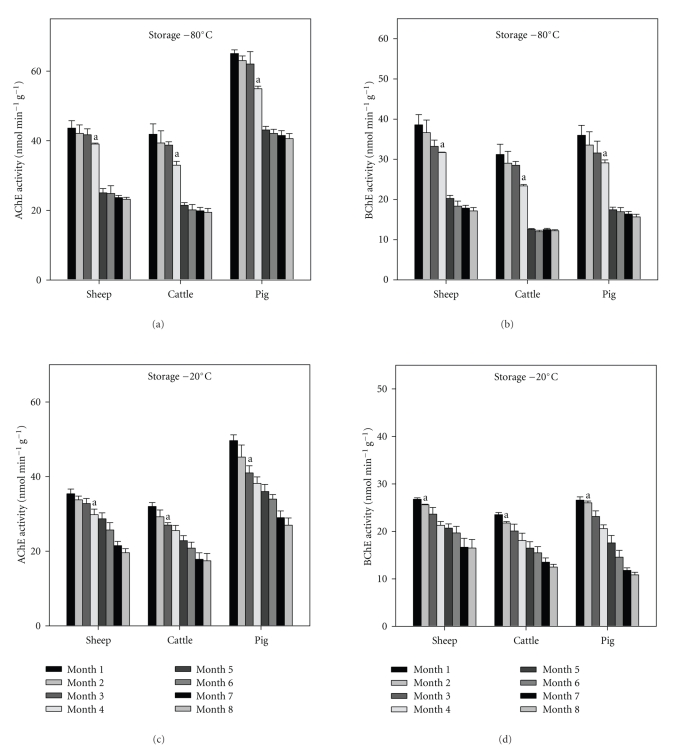
AChE and BChE activities in muscle for sheep, cattle and pigs using freezing at −80°C ((a) and (c)) and −20°C ((b) and (d)). Data are expressed as the mean ± SE (*n* = 10 of each animals). The letter in the column is significantly different (ANOVA, *P* < .05).

**Figure 7:**
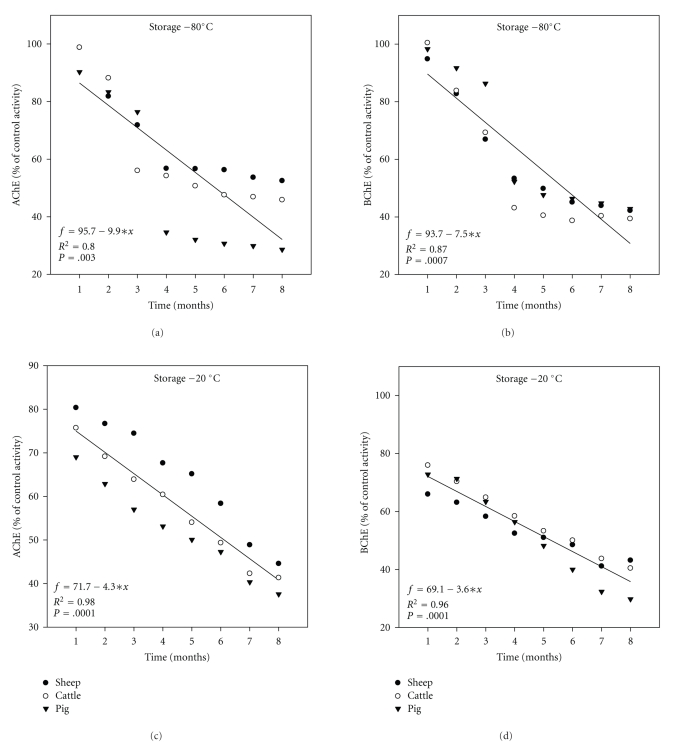
Regression analysis of ChE (% of control activities) over time in muscle freezing at −80°C for sheep, cattle, and pigs. Key for the figure is listed under Figure 2.

**Figure 8:**
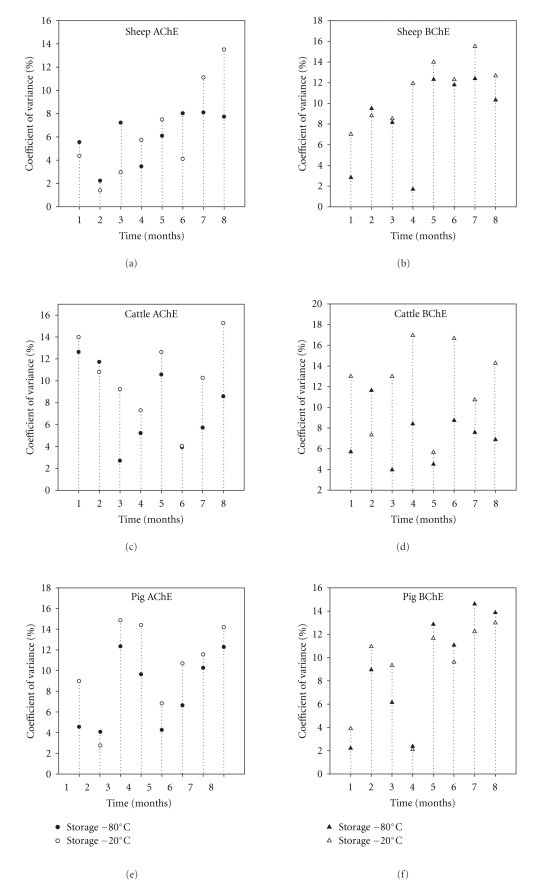
Percentage coefficient of variance between freezing at −80°C and −20°C for muscle sheep, cattle, and pigs in 8 months.

Freezing effects to AChE and BChE activities were also determined in muscle for sheep, cattle, and pigs using each of the 8 freezing times rates at −80°C and −20°C as described in Section 2 (Figures 6(a)–6(d)). In all cases (sheep, cattle, and pigs using both freezing methods), AChE activity was higher in muscle than BChE activity (Figures 6(a)–6(d)). In all cases (sheep, cattle, and pigs using both freezing methods), the freezing at −80°C showed a significant decrease after 3 months for AChE and BChE (Figures 6(a) and 6(b)). Freezing in all cases (sheep, cattle, and pigs using both freezing methods) at −20°C showed a significant decrease after 1 month for BChE (Figure 6(d)), while for AChE it showed a significant decrease after 2 months for cattle and pigs with the exception of sheep after 3 months (Figure 6(c)). Again linear regression of mean ChE activities was seen in muscle after 8 months of freezing (Figures 7(a)–7(d)). The *R* ^2^ values tended to be very high in case of AChE activity at −20°C (*R* ^2^ = 0.98, *P* = .0001; Figure 7(c)). Again taking the overall data set, there was a significant correlation between both AChE and BChE activities measured by −80°C and −20°C freezing (Pearson*'*s correlation coefficient = 0.43, *P* < .0001; Figure 3(b)), and again, the %CV values for each month were generally higher (35 out of 48 sets of data) using the −20°C compared to the −80°C freezing (Figures 8(a)–8(f)). Bland and Altman plot of the ratio of the freezing at −80°C and −20°C has shown the mean differences between the two freezing methods to be 1.5, and SD was 32.5 and −28.9 for upper and lower limits, respectively (Figure 5(b)). 

## 4. Discussion

The widespread use of organophosphorus and carbamate pesticides and the dangers associated with their application have resulted in cholinesterase (ChE) activities being used as biomarkers of both exposure to and effect of these pesticides [2]. As noted by Wilson et al. [2], determination of ChE activities may form the basis for the establishment of safe levels of such pesticides in food and in the environment. There are two freezing effects currently described for the measurement of ChE activities, the −20°C and −80°C. However, neither freezing effect has been validated for use either in tissues from other food animal or in other tissues. The present study was to investigate the effect of freezing (8 months) on activity of AChE and BChE for sheep, cattle, and pigs using modified Ellman method in liver and muscles as described in Section 2 (Figures 1 and 6). In all cases the results from our study are shown a significant decreases of AChE and BChE at −80°C after 6 months in liver (Figures 1(a) and 1(b)). In contrast, with muscle we found significant after 3 months (Figures 6(a) and 6(b)). This is in agreement with the work of Kirby et al. [20], who found no changes or loss in ChE activities for 4 months in freezing at −80°C for flounder muscle tissue. Nigg and Knaak [21], who observed a little change in human plasma BChE activity when freezing at −70°C after 10 times of frozen and thawing. Other than, ChE activities of fish brain tissue freezing at −68°C and −70°C for up to 55 days and 5 months, respectively did not differ significantly [14, 21]. In general freezing at −20°C showed significant decreases in all cases (sheep, cattle, and pigs for liver and muscle) after 1–3 months (Figures 1 and 6). This is in agreement with Crane et al. [22], who observed that plasma and erythrocyte ChE activities using freezing at −20°C remain stable after 6 weeks. This is in contrast with the work of Nigg and Knaak [21], who observed using freezing at −20°C for 14 months without significance loss of plasma BChE activity. Panteghini et al. [23], observed human plasma ChE activities to be stable for several months and years using freezing at −20°C. However, these authors used a human blood plasma measuring instead tissues of food animals. This factor may explain the apparent difference in activity. There is a 30% loss in BChE activity using freezing at −20°C in human serum, while there is no loss in AChE at storage for one year [24, 25]. And there is a 23% decrease in up to 6 months in sheep AChE activity using freezing at −20°C in whole blood and a 9% decrease of whole blood from dog using freezing at −20°C [26]. Morán and G*ό*mez-Ramos [27], who explained that some loss of AChE activity is due to particularly the G_4_ molecular form of the enzyme, which has been described in unfixed human brain tissue, stored frozen at −20°C for 4 weeks. 

Additionally, there is a great variety of freezing degrees that can be found among different laboratories, for example, there were no changes in ChE activities when stored more than ten years at lower than 4°C [28], and a10% decrease after 2 months in bovine erythrocyte ChE as well as a 95% decrease at 37°C for 4 days [29]. Furthermore, Balland et al. [30] found that ChE loses 15% of its activity after 240 days of storage at room temperature; additionally he reported that freezing for 1 h at −40°C and −196°C did not affect ChE activities in plasma and stored samples. High correlation coefficient was seen after 8 months of freezing at −80°C and −20°C in the liver and muscle (Figures 2 and 7). One objective of the present study was to investigate whether the frozen animal product had effect on activity of ChE [30]. 

Linear regression of mean ChE activity was observed in all individual samples on months of freezing at −80°C and −20°C (Figures 3(a) and 3(b)). The regression is used in present study to find the line that best predicts *y* (% control ChE activities) from *x* (months). The mean differences between the two freezing methods are plotted by Bland and Altman plot and were seen only in muscle storage clinically important the mean less than ±1.96 (Figure 5(b)). With regards to precision of the both freezing methods, they showed higher coefficient of variance (%CV) values using freezing at −20°C compared with freezing at −80°C (less than 13% and less than 15.2% for freezing at −80°C and −20°C, resp. for liver) and (less than 12.8% and less than 16.9% for freezing at −80°C and −20°C, resp. for muscle) (Figures 4 and 8); therefore freezing at −80°C provides better precision than freezing at −20°C in muscle and liver for sheep and cattle. Finally, it was noticed that the decreases of ChE inhibition levels after freezing were broadly similar to those found in the original analysis and, therefore, long-term freezing could still be used as an option during monitoring programmes, especially where samples are not allowed to thaw during storage.

## 5. Conclusions

This is the first study that provided original data concerning freezing effects for AChE and BChE activities in food animals. In general, using freezing at −80°C in all animals (sheep, cattle, and pigs), there is a significant inhibition after 6 months in liver and 3 months in muscle. While liver extracts using freezing at −20°C in all animals showed a significant decrease in AChE after 3 months with the exception of sheep. However, in general there are significant differences in BChE in muscle using freezing at −20°C after 1 month and in AChE after 2 months with exception of sheep AChE after 3 months. Despite this, further studies in different laboratories are necessary in order to improve our knowledge about this very interesting enzyme as a potential biochemical marker for intoxication.
